# Chemical Composition and Biological Activities of Geopropolis From Four *Melipona* Species From the Brazilian Atlantic Forest

**DOI:** 10.1002/cbdv.71615

**Published:** 2026-08-25

**Authors:** Ariane Pinheiro Cruz Bergamini, Antônio Domingos de Sousa Júnior, Flavia Vitorino de Araújo Porto, Iana Soares Pessoa, Thiago Antonio de Sousa Cutrim, Victor Paulo Mesquita Aragão, Victor da Rocha Fonseca, Wanderson Romão, Denise Coutinho Endringer, Rodrigo Scherer, Marcio Fronza

**Affiliations:** ^1^ Programa de Pós‐Graduação em Ciências Farmacêuticas Universidade Vila Velha Vila Velha Espirito Santo Brazil; ^2^ Petroleomics and Forensic Laboratory Universidade Federal do Espírito Santo (UFES) Vitória Espirito Santo Brazil

**Keywords:** antioxidants, antifungal agents, geopropolis, *melipona*, stingless bees

## Abstract

Geopropolis, a complex material produced by stingless bees from plant resins and soil components, represents a chemically diverse and underexplored source of natural products. This study investigated the chemical composition and biological activities of geopropolis from four *Melipona* species collected in the Brazilian Atlantic Forest during the dry and rainy seasons. Hydroalcoholic extracts were quantified for total phenolics, flavonoids, tannins, and characterized by electrospray ionization Fourier transform ion cyclotron resonance mass spectrometry (ESI(‐)‐FT‐ICR MS), allowing the tentative assignment of 56 compounds, mainly phenolic acids, flavonoids, and terpenoids. Antioxidant activity was evaluated using DPPH and ABTS assays, anti‐inflammatory activity by measuring nitric oxide, superoxide anion, and TNF‐α production in LPS‐stimulated macrophages, and antifungal activity against selected pathogenic strains. The extracts exhibited significant interspecific variation in phenolic composition and antioxidant activity (*p* < 0.05), with *Melipona bicolor* showing the highest antioxidant capacity, reflected by the lowest DPPH and ABTS IC_50_ values. Geopropolis extracts also significantly reduced nitric oxide, superoxide anion, and TNF‐α production (*p* < 0.05) and displayed selective antifungal activity. Seasonal variation influenced metabolite composition and biological activities, providing new insights into the chemical diversity and biological potential of geopropolis from native Atlantic Forest stingless bees.

## Graphical abstract

Geopropolis from four *Melipona* species collected in the Brazilian Atlantic Forest during the dry and rainy seasons was chemically characterized by ESI(‐)‐FT‐ICR MS and evaluated for antioxidant, anti‐inflammatory, and antifungal activities, demonstrating species‐ and season‐dependent variation in its chemical composition and biological properties.

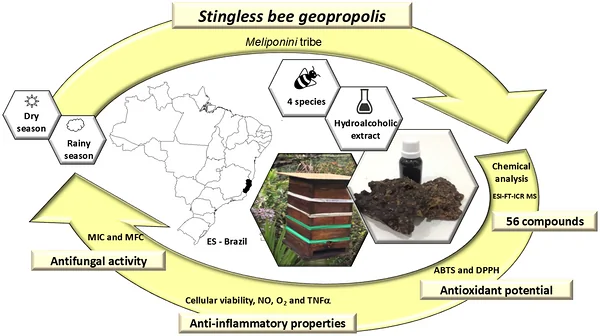

**Abbreviations**
ABTS: 2,2′‐Azino‐bis (3‐ethylbenzothiazoline‐6‐ sulfonic acid)
AMU: atomic mass units
BHT: Butil Hidroxi Toluol
DBE: double bond equivalents
DMSO: Dimethyl sulfoxide
DPPH: 2,2‐Diphenyl‐1‐picrylhydrazyl
ELISA: Enzyme Linked Immuno Sorbent Assay
(ESI(‐)‐FT‐ICR MS): Electrospray ionization Fourier transform ion cyclotron resonance mass spectrometry
GAE: gallic acid equivalents
HPLC: high‐performance liquid chromatography
HRMS: high‐resolution mass spectrometry
IC_50_
: Half maximal inhibitory concentration
LPS: Lipopolysaccharide
MB: *Melipona bicolor*
MC: *Melipona capixaba*
MDA: malondialdehyde
MM: *Melipona mondury*
MQ: *Melipona quadrifasciata*
MTT: 3‐(4,5‐Dimethylthiazol‐2‐yl)‐2,5‐Diphenyltetrazolium Bromide
NMR: nuclear magnetic resonance
NO: Nitric oxide
PVPP: polyvinylpolypyrrolidone
QE: quercetin equivalent
SD: Standard deviation
TNFα: Tumor necrosis factor aplha

## 1 Introduction

Brazilian biodiversity is widely recognized for its remarkable richness and the large number of species that continue to attract scientific interest [1]. Within this context, stingless bees have received increasing attention due to their ability to produce chemically complex materials with diverse biological properties [2, 3, 4]. These bees belong to the order Hymenoptera, family Apidae, and tribe *Meliponini*, and are distributed across tropical and subtropical regions worldwide [5]. Brazil harbors one of the greatest diversities of stingless bees, with 244 described species, including 39 species reported in the Atlantic Forest of Espírito Santo State [5, 6]. Among them are *Melipona (Eomelipona) bicolor*, *Melipona (Michmelia) capixaba*, *Melipona (Michmelia) mondury*, and *Melipona (Melipona) quadrifasciata* all of which are known to produce geopropolis [7, 8]. Notably, MC is an endemic species restricted to the state of Espírito Santo, Brazil, and previous studies involving MC and MB have focused only on their genetics, morphology, and behavioral characteristics [7, 9, 10, 11].

Geopropolis is a resinous material produced by stingless bees that contributes to nest structure and provides protection against biological and environmental threats. Unlike propolis produced by honeybees, geopropolis contains a complex mixture of bee salivary secretions, wax, plant resins, and soil, silt, and/or sand particles [12]. Its chemical composition is strongly influenced by botanical origin, geographic location, seasonality, and bee species, resulting in marked variability in metabolite profiles [13, 14].

Previous studies have shown that stingless bee geopropolis contains a wide variety of bioactive compounds, including phenylpropanoids, flavonoids, phenolic compounds, tannins, terpenes, saponins, alkaloids, essential oils, fatty acids, and sugars [3, 15]. Among these constituents, phenolic compounds, especially flavonoids, like quercetin and kaempferol, are widely associated with antioxidant activity. Their chemical structure, characterized by the presence of one or more hydroxyl group, contributes to oxidative stability by scavenging free radicals responsible for oxidative stress [3]. Accordingly, the antioxidant activity of ethanolic extracts of stingless bee propolis has been positively correlated with their phenolic and flavonoid contents [16].

Recent reviews have highlighted the broad spectrum of biological activities attributed to *Meliponini* geopropolis [3, 4, 15]. These activities vary according to extraction procedures, biological assays, sampling period, and bee species. Using different experimental models, stingless bee propolis extracts have demonstrated free radical scavenging activity comparable to that of well‐known antioxidant compounds commonly used as positive controls, such as gallic acid, ellagic acid, ascorbic acid, and butylated hydroxytoluene (BHT) [17, 18, 19]. In addition, geopropolis extracts have been reported to protect against oxidative hemolysis and reduce malondialdehyde (MDA) levels, a recognized biomarker of lipid peroxidation induced by oxidative stress. However, Bonamigo et al. (2017) [20] observed that aerotropolis from *Plebeia droryana* failed to inhibit MDA production induced by 2,2'‐azobis(2‐amidinopropane) dihydrochloride, suggesting that antioxidant effects may vary according to the chemical composition of the extract.

Besides their antioxidant properties, geopropolis extracts have demonstrated considerable antimicrobial activity [21, 22]. This activity appears to involve two complementary mechanisms: direct inhibition of microbial pathogens through interference with essential metabolic processes and indirect enhancement of the host immune response, thereby improving the ability to eliminate invading microorganisms and limit tissue damage [23]. These findings collectively support the pharmacological potential of geopropolis as a natural source of biologically active compounds.

Despite growing interest in stingless bee geopropolis, important knowledge gaps remain. Most studies investigating the chemical composition and biological activities of geopropolis have focused on species from other Brazilian biomes or geographical regions. Information regarding Atlantic Forest *Melipona* species remains scarce, particularly for MC and MB, whose geopropolis has received little scientific attention. Furthermore, no comparative study has systematically assessed the influence of seasonal variation on both chemical composition and multiple biological activities of geopropolis produced by different *Melipona* species from the Atlantic Forest. Consequently, important questions remain unanswered regarding species‐specific chemical markers, seasonal changes in metabolite production, and their potential relationships with biological activity. This knowledge gap limits our understanding of the chemical diversity and pharmacological potential of geopropolis produced by Atlantic Forest stingless bees.

Based on the recognized influence of botanical origin, bee species, and environmental conditions on geopropolis composition, we hypothesized that geopropolis produced by different Atlantic Forest *Melipona* species would exhibit species‐specific and season‐dependent metabolite profiles, resulting in distinct antioxidant, anti‐inflammatory, and antifungal activities.

Therefore, this study aimed to characterize the chemical composition of hydroalcoholic extracts of geopropolis produced by four *Melipona* species from the Atlantic Forest of Espírito Santo, Brazil, using high‐resolution ESI‐FT‐ICR MS, and to investigate their antioxidant, anti‐inflammatory, and antifungal properties. In contrast to previous studies focused on individual species or single sampling periods, we also evaluated the influence of seasonal variation on both metabolite composition and biological activities across multiple *Melipona* species. This approach provides new insights into the chemical diversity and pharmacological potential of geopropolis from endemic and poorly explored stingless bee species.

## 2 Results

The highest yields of geopropolis extracts were obtained during the dry season from MQ, MM, MC, and MB bees, with yields of 27.3%, 27.4%, 24.7%, and 24.2% (w/w), respectively. In contrast, yields during the rainy season were 16.6%, 24.9%, 21.6%, and 12.3% (w/w) for MQ, MM, MC, and MB, respectively.

**FIGURE 1:**
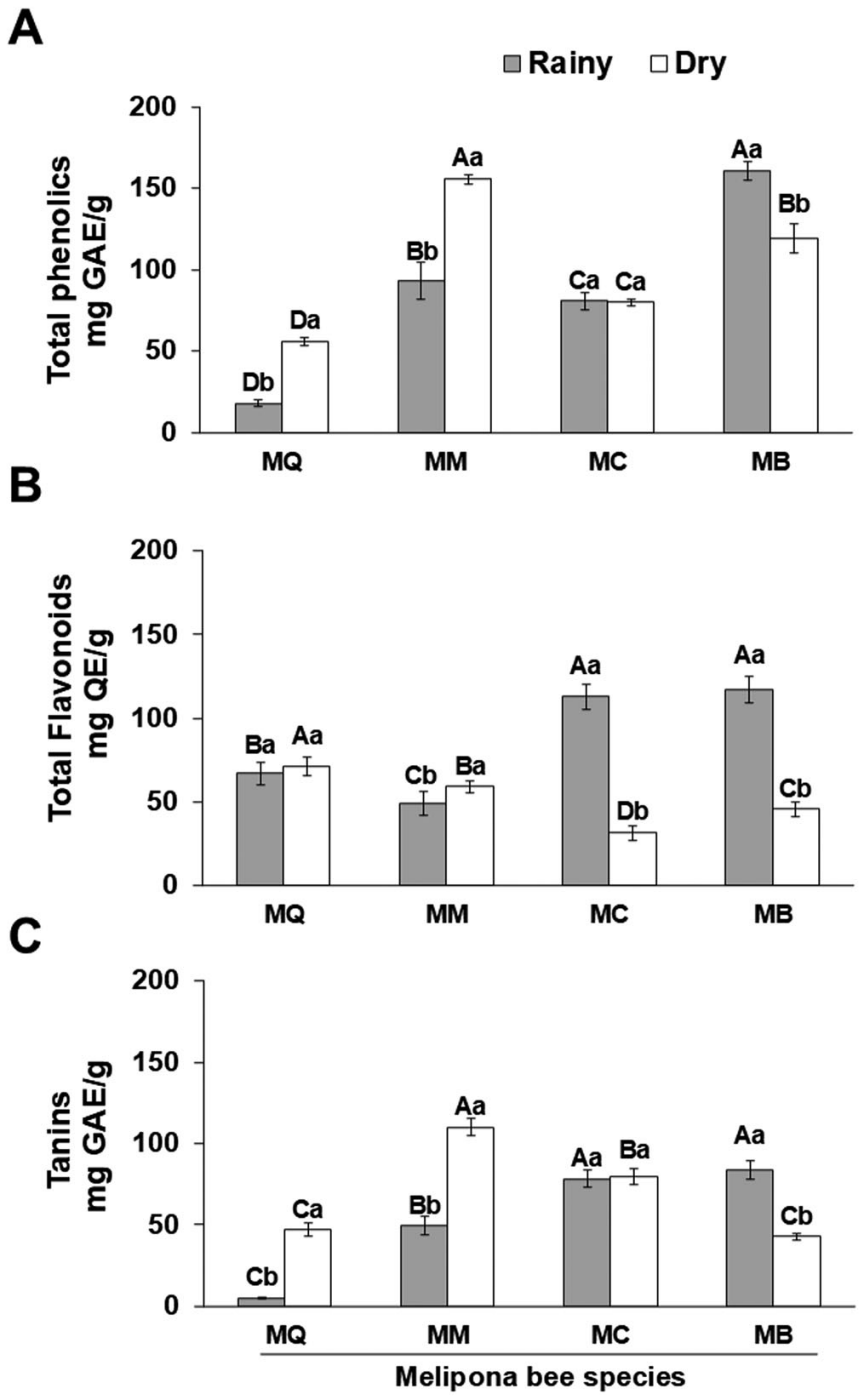
Total phenolic (A), flavonoid (B), and tannin (C) contents of geopropolis extracts obtained from MQ, MM, MC, and MB collected during the rainy and dry seasons. Total phenolics and tannins are expressed as milligram gallic acid equivalents (GAE)/g dry extract, whereas flavonoids are expressed as milligram quercetin equivalents (QE)/g dry extract. Data are presented as mean ± standard deviation (SD) from three independent experiments performed in triplicate. Different uppercase letters indicate significant differences among bee species within the same season, whereas different lowercase letters indicate significant differences between seasons for the same bee species (two‐way ANOVA followed by Tukey's post hoc test, *p* < 0.05).

The contents of total phenolics, flavonoids, and tannins varied significantly among *Melipona* species and between collection seasons (*p* < 0.05), demonstrating that both bee species and seasonality influence the chemical composition of geopropolis (Figure 1).

Regarding total phenolic content (Figure 1A), MB exhibited the highest concentration during the rainy season (160.68 ± 5.70 mg GAE/g), whereas MM showed the highest phenolic content in the dry season (155.68 ± 2.80 mg GAE/g). In contrast, MQ consistently presented the lowest phenolic levels in both seasons. Notably, MC was the only species that did not exhibit significant seasonal variation in total phenolic content, indicating a relatively stable chemical composition throughout the year. The phenolic concentrations determined for MM and MQ were consistent with previous reports for these species [14, 24].

A similar species‐dependent pattern was observed for flavonoids (Figure 1B). During the rainy season, MB and MC contained the highest flavonoid concentrations, whereas, MM exhibited significantly lower levels. Seasonal variation markedly affected flavonoid accumulation in most species, with substantial reductions observed during the dry season for MB, MC, and MM. Conversely, MQ was the only species whose flavonoid content remained statistically unchanged between seasons. Furthermore, the flavonoid concentration observed for MQ during the dry season exceeded values previously reported for *Melipona quadrifasciata anthidioides* [25]. To our knowledge, comparable flavonoid data are not available for the remaining species.

Tannin contents also differed significantly among species and seasons (Figure 1C). During the rainy season, the highest concentrations were found in MB and MC, whereas MQ exhibited only trace amounts. Seasonal variation strongly influenced tannin accumulation in MB, MM, and MQ, while MC again showed no significant seasonal differences, reinforcing the stability of its phenolic profile. To the best of our knowledge, this is the first study reporting tannin quantification in geopropolis produced by these *Melipona* species, since comparable data for *Meliponini* geopropolis are currently unavailable in the literature.

High‐resolution ESI(‐)‐FT‐ICR MS analysis enabled the tentative annotation of 56 metabolites in the geopropolis extracts based on accurate mass measurements, molecular formula assignment, double bond equivalent (DBE) values, comparison with previously reported compounds, and mass errors lower than 10 ppm (Table S1). Overall, the mass spectral profiles were qualitatively similar among the four *Melipona* species and collection seasons, differing mainly in the relative abundance of individual ions (Figure S1).

The identified metabolites were grouped into seven major chemical classes (Table 1), with flavonoids representing the most abundant class (25 Compounds), followed by phenolic compounds (8 Compounds), phenylpropanoids (7 Compounds), fatty acids (7 Compounds), other metabolites (5 Compounds), terpenoids (2 Compounds), and xanthones (2 Compounds). This predominance of phenolic‐derived metabolites is consistent with previous reports describing geopropolis as a rich source of antioxidant phytochemicals [2, 26, 27].

**TABLE 1:** Overview of the chemical classes tentatively annotated in geopropolis extracts from four *Melipona* species by ESI(‐)‐FT‐ICR MS.

Chemical class	Representative metabolites^*^	Number of compounds
Phenolic compounds	Quinic acid, syringic acid, ellagic acid, monogalloylglucose, and digalloylshikimic acid	8
Flavonoids	Chrysin, apigenin, naringenin, kaempferol, quercetin, rutin, naringin, isoquercetin, quercitrin	25
Phenylpropanoids	Ferulic acid, sinapic acid, chlorogenic acid, rosmarinic acid, caffeoylquinic acid	7
Terpenoids	Carnosol, sesquiterpene hydrocarbons (e.g., caryophyllene‐type compounds)	2
Fatty acids	Palmitic, linolenic, linoleic, oleic, and stearic acids	7
Xanthones	Mangostin, mangiferin	2
Other compounds	Glucose, retinol, nemorosone/propolone A	5
Total	—	56

* Only representative metabolites are shown. The complete list of tentatively annotated compounds, molecular formulas, mass errors, DBE values, and occurrence among samples is presented in Table S1.

Phenolic compounds included quinic acid, syringic acid, monogalloylglucose, digalloylshikimic acid, syringaldehyde, sinapaldehyde, ellagic acid, and its dimer. Several of these metabolites have previously been associated with antioxidant properties and have been reported in stingless bee geopropolis from other Brazilian regions [2, 17, 19, 20, 26, 27, 28, 29, 30].

Flavonoids constituted the largest chemical group detected and comprised flavones (e.g., chrysin and apigenin), flavonols (e.g., kaempferol, quercetin, myricetin, and isorhamnetin), flavanones (e.g., naringenin and taxifolin), flavan‐3‐ols (catechin/epicatechin), prenylated flavonoids, and glycosylated derivatives, such as rutin, naringin, isoquercetin, quercitrin, and quercetin diglucoside. The abundance and structural diversity of flavonoids likely contribute to the pronounced antioxidant and anti‐inflammatory activities observed in the biological assays [2, 27, 29, 31, 32].

Phenylpropanoids were represented mainly by cinnamic acid derivatives, including ferulic acid, sinapic acid, prenylated caffeic acid derivatives, chlorogenic acid, rosmarinic acid, and caffeoylquinic acid. These metabolites have previously been associated with antioxidant, antimicrobial, and anti‐inflammatory activities in bee products [2, 20].

The terpene fraction consisted predominantly of sesquiterpene hydrocarbons together with the oxygenated diterpenoid carnosol, whereas the lipid fraction comprised gluconic acid, palmitic, linolenic, linoleic, oleic, and stearic acids. In addition, two xanthones (mangostin and mangiferin) and a limited number of other metabolites, including glucose, retinol, and nemorosone/propolone A, were tentatively annotated [2, 17].

Because ESI‐FT‐ICR MS provides highly accurate molecular mass information, but does not unequivocally resolve structural isomers, compounds sharing identical elemental compositions (e.g., quercetin/tricetin, kaempferol/luteolin, catechin/epicatechin, and chlorogenic acid/caffeoylquinic acid) were conservatively reported as alternative tentative annotations. Therefore, compound assignments should be interpreted as putative annotations based on accurate mass and literature comparison, while unequivocal structural confirmation would require complementary MS/MS fragmentation analyses and/or authentic reference standards. Overall, the predominance of phenolic compounds, flavonoids, and phenylpropanoids agrees well with the high antioxidant and anti‐inflammatory activities observed for the geopropolis extracts, suggesting that these metabolite classes are major contributors to the biological properties of geopropolis from Atlantic Forest *Melipona* species.

**FIGURE 2:**
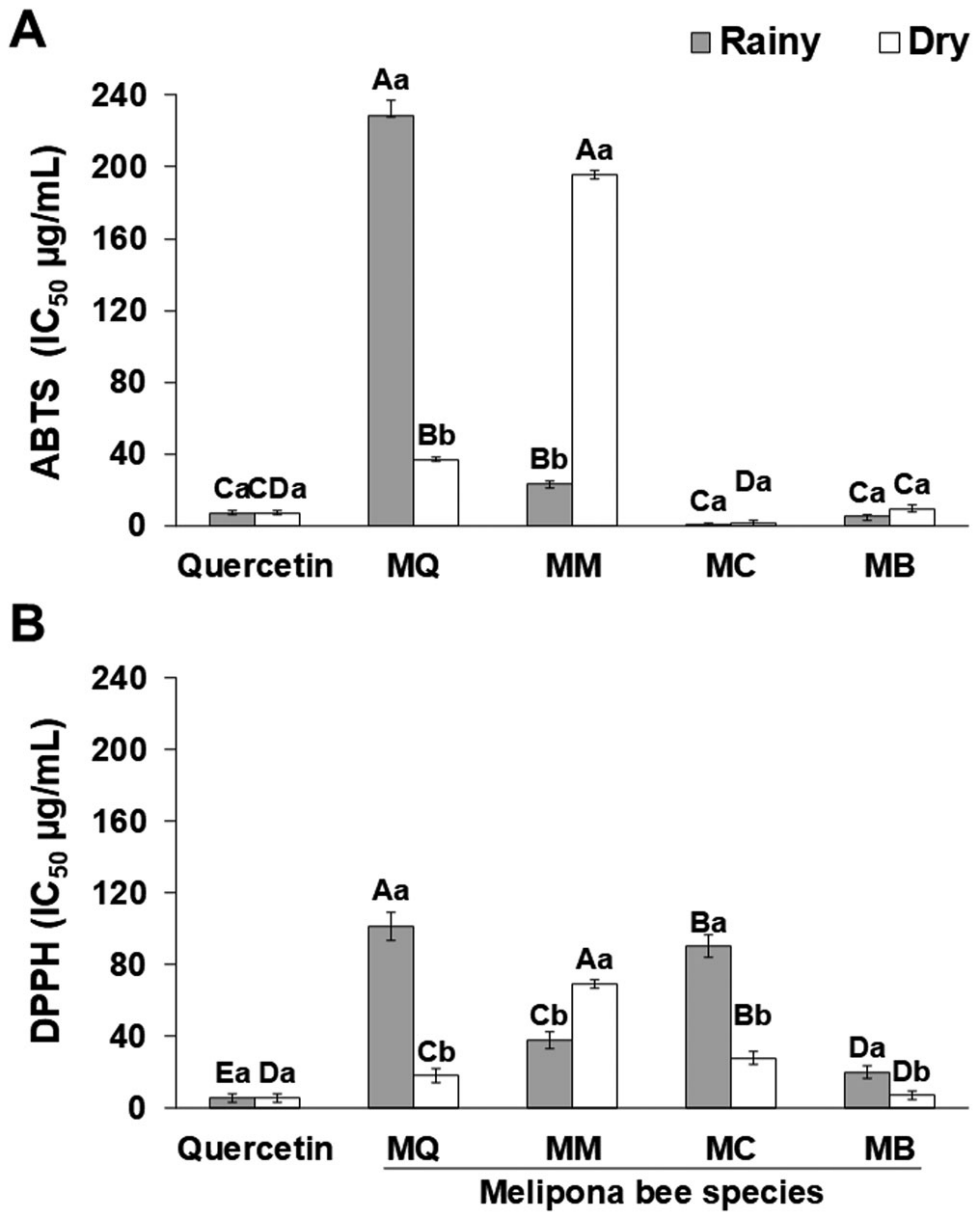
Antioxidant activities of geopropolis extracts obtained from MQ, MM, MC, and MB collected during the rainy and dry seasons. Antioxidant capacity was evaluated by the ABTS radical scavenging assay (A) and the DPPH radical scavenging assay (B). Results are expressed as IC_50_ values (µg/mL) and presented as mean ± standard deviation (SD) from three independent experiments performed in triplicate. Different uppercase letters indicate significant differences among bee species within the same season, whereas different lowercase letters indicate significant differences between seasons for the same bee species (two‐way ANOVA followed by Tukey's test, *p* < 0.05).

Considering the predominance of phenolic compounds and flavonoids identified by ESI(‐)‐FT‐ICR MS, the antioxidant potential of the geopropolis extracts was evaluated using the ABTS and DPPH radical scavenging assays (Figure 2).

Significant differences in antioxidant activity were observed among the four *Melipona* species and between collection seasons (*p* < 0.05), demonstrating that both bee species and seasonality influenced the radical scavenging capacity of the extracts. Overall, MB exhibited the highest antioxidant activity in both assays, with the lowest IC_50_ values irrespective of the collection season. In the DPPH assay (Figure 2B), MB geopropolis presented IC_50_ values of 20.03 ± 1.93 µg/mL (rainy season) and 7.02 ± 0.21 µg/mL (dry season), the latter being statistically comparable to the positive control quercetin. In contrast, marked seasonal effects were observed for MQ and MM, indicating that environmental conditions strongly influence the antioxidant properties of geopropolis in these species.

Similar trends were observed in the ABTS assay (Figure 2A). Geopropolis from MB and MC, collected during both rainy and dry seasons, exhibited radical scavenging activity comparable to that of quercetin, whereas MQ and MM displayed greater seasonal variation. The high antioxidant activity observed for MB and MC is consistent with the predominance of phenolic compounds and flavonoids identified in these extracts and represent the first report describing the antioxidant potential of geopropolis from these Atlantic Forest stingless bee species.

The effects of geopropolis extracts on cell viability were evaluated in RAW 264.7 macrophages and L929 fibroblast using the MTT colorimetric assay. None of the extracts exhibited cytotoxicity at concentrations ranging from 0.1 to 20 µg/mL in either cell line. Thus, subsequent anti‐inflammatory assays were performed using this non‐cytotoxic concentration range to ensure that the observed effects were not attributable to reduced cell viability.

**FIGURE 3:**
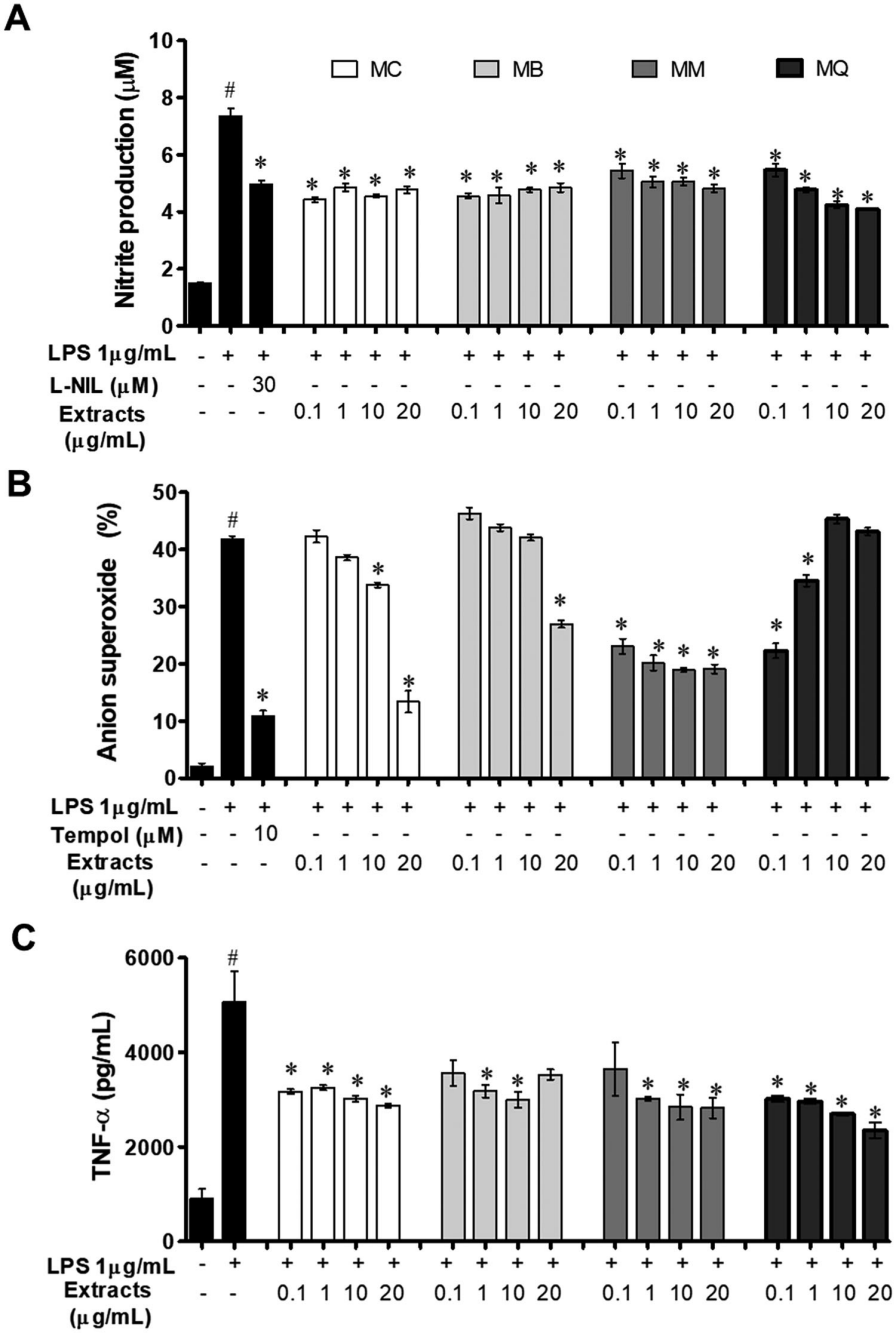
In vitro anti‐inflammatory potencial of geopropolis extracts of MC, MB, MM, and MQ evaluated in LPS‐stimulated RAW 264.7 macrophages. (A) Nitric oxide production, (B) superoxide anion O_2_•^−^; and (C) TNF‐α production. Results are presented as mean ± SD from three independent experiments performed in triplicate. # indicates a significant difference between the untreated control and the LPS‐stimulated control (*p* < 0.05). * indicates a significant difference compared with the LPS‐treated control (one‐way ANOVA followed by Tukey's post hoc test, *p* < 0.05).

The anti‐inflammatory potential of geopropolis extracts was investigated by evaluating the production of hey inflammation mediators, including nitric oxide (NO), superoxide anion (O_2_•^−^), and tumor necrosis factor‐α (TNF‐α), in LPS‐stimulated RAW 264.7 macrophages (Figure 3).

LPS stimulation markedly increased nitrite production compared with the untreated control, confirming macrophage activation (Figure 3A). Treatment with geopropolis extracts from all four *Melipona* species significantly reduced NO production at all tested concentrations (0.1–20 µg/mL) (*p* < 0.05). Although the inhibitory effect was consistently observed for all extracts, no clear dose‐dependent response was detected, suggesting that NO inhibition was achieved even at the lowest concentration evaluated.

The inhibitory effects on intracellular superoxide anion production are shown in Figure 3B. In contrast to the NO assay, the reduction in O_2_•^−^ production exhibited a concentration‐dependent trend for most extracts. The greatest inhibition was observed at 20 µg/mL, with the exception of MQ, which showed only a modest reduction across the tested concentrations. Among the evaluated samples, the geopropolis extract from MC demonstrated the strongest inhibitory activity, reaching levels comparable to those obtained with the positive control Tempol at the highest concentration tested.

The production of TNF‐α was also significantly increased following LPS stimulation, confirming the establishment of an inflammatory response (Figure 3C). Treatment with geopropolis extracts significantly reduced TNF‐α secretion, particularly at 20 µg/mL, regardless of the *Melipona* species evaluated. Although the magnitude of inhibition varied among species, all extracts attenuated the production of this pro‐inflammatory cytokine compared with the LPS‐treated control.

Overall, the ability of the geopropolis extracts to simultaneously reduce the production of NO, superoxide anion, and TNF‐α demonstrates a consistent anti‐inflammatory profile. These findings are in agreement with the chemical characterization, which revealed the predominance of phenolic compounds, flavonoids, and phenylpropanoids, classes of metabolites widely recognized for their antioxidant and immunomodulatory properties.

The antifungal activity of the geopropolis extracts was evaluated against six clinically relevant fungal strains by determining the minimum inhibitory concentration (MIC) and minimum fungicidal concentration (MFC) (Table 2).

**TABLE 2:** Antifungal activities of geopropolis extracts from MQ, MM, MC, and MB collected during the rainy and dry seasons. Antifungal activity was determined by the minimum inhibitory concentration (MIC) and minimum fungicidal concentration (MFC) against reference fungal strains. Results are expressed as µg/mL.

Bee species	Season	*Malassezia furfur* *CCT 1349*		*Trichophyton rubrum* *CBS 392.58*		*Candida albicans ATCC 10231*		*Candida albicans* ATCC 90029		*Candida parapsilosis* ATCC 22019		*Aspergillus fumigatus* *ATCC 16913*	
		MIC	MFC	MIC	MFC	MIC	MFC	MIC	MFC	MIC	MFC	MIC	MFC
MQ	dry	1024	>1024	256	256	>1024	>1024	>1024	>1024	>1024	>1024	1024	>1024
	rainy	>1024	>1024	512	>1024	>1024	>1024	>1024	>1024	>1024	>1024	>1024	>1024
MM	dry	>1024	>1024	1024	>1024	>1024	>1024	>1024	>1024	>1024	>1024	>1024	>1024
	rainy	>1024	>1024	1024	>1024	>1024	>1024	>1024	>1024	>1024	>1024	>1024	>1024
MC	dry	>1024	>1024	1024	>1024	>1024	>1024	>1024	>1024	>1024	>1024	>1024	>1024
	rainy	>1024	>1024	1024	>1024	>1024	>1024	>1024	>1024	>1024	>1024	>1024	>1024
MB	dry	512	>1024	>1024	>1024	>1024	>1024	>1024	>1024	>1024	>1024	>1024	>1024
	rainy	>1024	>1024	1024	>1024	>1024	>1024	>1024	>1024	>1024	>1024	>1024	>1024
*Fluconazole*		2	>1024	>1024	16	0,5	4	0,5	4	2	4	128	>1024
*Amphotericin B*		—	—	0.06	0.06	0.06	0.06	0.25	0.25	0.25	0.25	—	—

(–), not determined.

Overall, the geopropolis extracts exhibited selective antifungal activity, with susceptibility varying according to the fungal species and the *Melipona* source. Among the evaluated samples, only extracts from MQ and MB inhibited fungal growth at the concentrations tested (≤1024 µg/mL). The strongest activity was observed for the dry‐season MQ extract against *Trichophyton rubrum*, which exhibited both fungistatic and fungicidal activity (MIC = MFC = 256 µg/mL). The same extract also inhibited the growth of *Malassezia furfur* (MIC = 1024 µg/mL), although no fungicidal effect was detected. Geopropolis from *M. bicolor* collected during the dry season also inhibited *M. furfur* (MIC = 512 µg/mL), whereas extracts obtained during the rainy season and those from MM and MC showed no detectable antifungal activity against the tested strains. Likewise, none of the extracts inhibited the growth of *Candida albicans, Candida parapsilosis*, or *Aspergillus fumigatus* at the highest concentration evaluated. These findings indicate that the antifungal activity of Atlantic Forest geopropolis is species‐dependent and appears to be restricted to specific fungal pathogens, particularly dermatophytes and lipophilic yeasts.

## 3 Discussion

The growing interest in natural products derived from stingless bees has stimulated research into the chemical diversity and biological properties of geopropolis. Nevertheless, the chemical composition and pharmacological potential of geopropolis produced by Atlantic Forest *Melipona* species remain largely unexplored, particularly with respect to seasonal variation. In this context, the present study provides the first comprehensive characterization of the geopropolis produced by MC, MB, and MM, integrating high‐resolution mass spectrometry with the evaluation of antioxidant, anti‐inflammatory, and antifungal activities. The results demonstrate that both bee species and seasonality substantially influence the chemical composition and biological properties of geopropolis, reinforcing the importance of biodiversity as a determinant of natural product composition and bioactivity.

The extraction procedure adopted in this study was selected based on its efficiency in recovering phenolic compounds from propolis matrices. Several factors, including solvent composition, extraction temperature, extraction time, particle size, and extraction technique, directly influence the recovery of bioactive constituents. Hydroethanolic mixtures are widely recognized as the most efficient solvents for extracting phenolic compounds and flavonoids from propolis. Dos Santos et al. (2017) reported higher extraction yields using hydroalcoholic solvents than aqueous extraction for MQ and *Tetragonisca angustula* [24]. Likewise, Dutra et al. (2014) demonstrated satisfactory extraction efficiency using the same extraction protocol employed in the present study. Furthermore, ultrasound‐assisted extraction considerably reduces extraction time, while maintaining extraction efficiency, making it an appropriate strategy for recovering geopropolis metabolites [33].

High‐resolution ESI(‐)‐FT‐ICR MS enabled the tentative annotation of 56 metabolites belonging predominantly to flavonoids, phenolic compounds, phenylpropanoids, fatty acids, terpenoids, and xanthones. Although the relative abundance of these metabolites varied among species and seasons, the overall distribution of chemical classes remained remarkably similar. These findings suggest that Atlantic Forest geopropolis shares a conserved chemical framework, while exhibiting quantitative variations associated with bee species and environmental conditions. Because FT‐ICR MS provides accurate molecular formula assignment, but cannot unequivocally distinguish structural isomers or determine stereochemistry, compound identification should be regarded as tentative. Complementary analytical approaches, including chromatographic separation coupled with authentic standards and nuclear magnetic resonance spectroscopy will be necessary for complete structural elucidation.

The predominance of flavonoids and other phenolic compounds agrees with previous reports describing these metabolites as the principal bioactive constituents of stingless bee geopropolis [2, 26, 27, 28, 30]. Flavonoids, such as quercetin, kaempferol, rutin, catechin, naringenin, and apigenin are widely recognized for their ability to scavenge reactive oxygen species, modulate inflammatory mediators, and inhibit microbial growth. Likewise, phenylpropanoid derivatives, including caffeic, ferulic, chlorogenic, and rosmarinic acids, have been associated with antioxidant and anti‐inflammatory activities through hydrogen atom donation and modulation of redox‐sensitive biological processes [34]. Therefore, the simultaneous occurrence of these compounds likely contributes to the biological properties observed in the present study.

Among the less abundant metabolites, terpenoids, and xanthones deserve particular attention because of their broad pharmacological relevance. Terpenoids, such as carnosol have been associated with antioxidant, antimicrobial, and anti‐inflammatory activities, whereas sesquiterpenes identified in the extracts have frequently been reported in geopropolis from other stingless bee species [2, 27]. Likewise, Phenolic acids, hydrolyzable tannins, and antioxidant activity of geopropolis from the stingless bee *melipona*, two xanthones tentatively identified in the present study, have previously been reported in stingless bee products [35]. These metabolites exhibit multiple biological activities, including antioxidant, antimicrobial, antidiabetic, antitumor, and immunomodulatory properties, suggesting that they may contribute to the overall biological profile of geopropolis despite their relatively low abundance.

Seasonality emerged as one of the major factors influencing the chemical composition of geopropolis. Such variation is expected because the Atlantic Forest exhibits pronounced seasonal changes in flowering phenology, resin availability, rainfall, and temperature, all of which directly influence plant secondary metabolism. During periods of greater floral abundance, bees have access to a broader diversity of resin‐producing plants, whereas reduced resource availability during the dry season may alter resin selection and foraging behavior [36]. Consequently, the botanical origin of collected resins varies throughout the year, resulting in seasonal changes in the chemical composition of geopropolis. Similar ecological influences have previously been reported for stingless bee products from other Brazilian biomes [3, 4, 15]. In addition, species‐specific foraging preferences and resin selection behavior may further contribute to the distinct chemical profiles observed among *Melipona* species.

The considerable variation observed in phenolic, flavonoid, and tannin contents among species and seasons reinforces the need to consider botanical origin and seasonality during the standardization of stingless bee geopropolis. Currently, Brazilian legislation (IN/MAPA n° 3/2001) establishes quality parameters based exclusively on *Apis mellifera* propolis, requiring minimum concentrations of 2.5 mg/g flavonoids and 5.0 mg/g total phenolics. All geopropolis samples evaluated in the present study exceeded these minimum requirements, suggesting that these criteria may provide an initial reference for stingless bee geopropolis until specific regulatory standards become available.

The antioxidant capacity of geopropolis extracts varied significantly among species and between collection seasons. Among all samples, MB exhibited the lowest IC_50_ values in both DPPH and ABTS assays, indicating the greatest free radical scavenging capacity, since lower IC_50_ values correspond to higher antioxidant activity. This superior performance is likely associated with its higher concentrations of phenolic compounds and flavonoids together with the presence of metabolites, such as quercetin, kaempferol, rutin, mangiferin, and other phenolic derivatives known for their antioxidant properties [37]. Likewise, the enhanced antioxidant activity observed in samples collected during the rainy season may reflect seasonal changes in resin sources, leading to increased accumulation of redox‐active phenolic metabolites. Similar species‐ and season‐dependent variations have been described previously for stingless bee geopropolis [20]. Although DPPH and ABTS assays are widely used for screening antioxidant potential, they evaluate the chemical capacity of extracts to neutralize synthetic free radicals under controlled conditions and should not be interpreted as direct evidence of biological antioxidant efficacy. In living systems, antioxidant activity is influenced by multiple factors, including absorption, metabolism, cellular uptake, and interactions with endogenous antioxidant defense systems. Therefore, the present findings demonstrate the chemical antioxidant potential of Atlantic Forest geopropolis, but do not necessarily predict its biological antioxidant effects in vivo.

The geopropolis extracts also significantly reduced the production of nitric oxide, superoxide anion, and TNF‐α in LPS‐stimulated RAW 264.7 macrophages, supporting their anti‐inflammatory potential. These mediators play central roles in inflammatory responses and oxidative stress and are frequently used as biomarkers for the preliminary screening of anti‐inflammatory natural products [38]. The inhibitory effects observed in the present study are consistent with previous reports describing the anti‐inflammatory properties of geopropolis and propolis from stingless bees [3, 4, 15]. Nevertheless, the present study was designed to evaluate functional inflammatory markers and did not investigate the molecular mechanisms underlying these biological effects. Consequently, no conclusions can be drawn regarding the involvement of intracellular signaling pathways, such as NF‐κB, MAPKs, iNOS, COX‐2, or other regulatory mechanisms commonly associated with inflammatory responses. Future studies employing molecular and biochemical approaches will be required to clarify the signaling pathways responsible for the observed inhibition of inflammatory mediators.

The antifungal evaluation demonstrated selective rather than broad‐spectrum activity. Geopropolis from MQ and MB inhibited the growth of *Trichophyton rubrum* and *M. furfur*, whereas no activity was detected against *Candida* spp. or *A. fumigatus* within the tested concentration range. Similar selective antifungal activity has been reported for geopropolis from *Frieseomelitta longipes*, *Tetragonisca fiebrigi*, and *Melipona orbignyi* [13, 16, 17, 18, 39]. The differences observed among fungal species probably reflect variations in cell wall composition, membrane permeability, and susceptibility to specific phytochemicals present in geopropolis. Previous studies have proposed that propolis may inhibit fungal growth by interfering with enzymes, membrane integrity, and metabolic pathways essential for pathogen survival while simultaneously contributing to the maintenance of the host antioxidant status during infection [23]. However, the specific compounds responsible for the antifungal activity observed in the present study remain to be identified.

Overall, this work substantially expands current knowledge regarding the chemical diversity and biological properties of geopropolis produced by Atlantic Forest stingless bees. By demonstrating that both bee species and seasonality influence phytochemical composition and biological activity, the present study highlights the importance of considering ecological factors when investigating bee products. Although additional studies are still required to isolate individual bioactive compounds, confirm their structures, and elucidate their molecular mechanisms of action, the present findings provide a robust scientific basis for future pharmacological investigations and reinforce the biotechnological potential of Atlantic Forest geopropolis as a promising source of natural bioactive compounds.

## 4 Conclusions

This study provides the first characterization of the chemical composition and in vitro biological activities of geopropolis produced by four *Melipona* species native to the Brazilian Atlantic Forest. High levels of phenolics, flavonoids, and tannins were detected, and high‐resolution ESI(‐)‐FT‐ICR MS enabled the tentative annotation of compounds belonging to several phytochemical classes. The extracts also exhibited radical scavenging activity, reduced the production of nitric oxide, superoxide anion, and TNF‐α in LPS‐stimulated macrophages, and showed selective antifungal activity. In addition, seasonal variation influenced both the chemical profiles and the biological activities of the geopropolis samples.

Overall, these findings expand the current knowledge of the chemical diversity and biological potential of geopropolis from previously unexplored stingless bee species. However, since the present study was limited to chemical characterization and in vitro assays, further studies are required to confirm compound identities, elucidate the underlying mechanisms of action, and evaluate the pharmacokinetic, toxicological, and in vivo effects before considering potential therapeutic applications.

## 5 Experimental Section

### 5.1 Sample Collection

Geopropolis samples from the four selected stingless bee species of the tribe *Meliponini*‐ *Melipona* (Melipona) *quadrifasciata* (Lepeletier, 1836) (MQ), *Melipona* (Michmelia) *mondury* (Smith, 1863) (MM), *Melipona* (Michmelia) *capixaba* (Moure & Camargo, 1994) (MC), *Melipona* (Eomelipona) *bicolor* (Lepeletier, 1836) (MB)—were collected in Santa Maria Jetibá (20°05'52''S, 40°36'56''W), Espírito Santo, Brazil, during the dry season of 2019 (September) and the rainy season of 2020 (February). The samples were obtained directly from the hives by spatulation, placed in properly labeled plastic bags, protected from light, and stored at −20 °C until analysis.

### 5.2 Geopropolis Hydroalcoholic Extracts

Geopropolis samples collected from each stingless bee species were initially ground into a fine powder using a ball mill to ensure sample homogenization. The powdered material was then exhaustively extracted by ultrasound‐assisted maceration with 70% (v/v) ethanol at 40 °C, using a sample‐to‐solvent ratio of 1:10 (w/v), following the procedure described by Dutra et al. (2014) [14]. After extraction, the resulting hydroalcoholic extracts were filtered to remove insoluble residues, concentrated under reduced pressure using a rotary evaporator to remove the solvent, and subsequently lyophilized. The dried extracts were stored at −20 °C until further chemical and biological analyses.

### 5.3 Determination of Total Flavonoid, Phenolics, and Tannins Content

The total flavonoid content of the geopropolis extracts was determined using a spectrophotometric assay based on the formation of flavonoid‐aluminum chloride complexes, as previously described [37]. Briefly, the extracts were reacted with 10% (w/v) aluminum chloride solution, and the absorbance was measured at 415 nm using a SpectraMax 190 microplate spectrophotometer (Molecular Devices, Sunnyvale, CA, USA). Quantification was performed using a quercetin calibration curve (10‐200 µg/mL), and the results were expressed as milligrams of quercetin equivalents (QE) per gram of dry extract (mg QE/g).

The total phenolic and tannin contents were determined using the Folin–Ciocalteu colorimetric method [37]. A calibration curve was prepared using gallic acid (1–180 µg/mL), and absorbance was measured at 715 nm with the same spectrophotometer. Total phenolic content was calculated from the gallic acid calibration curve and expressed as milligrams of gallic acid equivalents (GAE) per gram of dry extract (mg GAE/g). Tannin content was estimated by precipitation of tannins with polyvinylpolypyrrolidone (PVPP), followed by determination of the remaining non‐tannin phenolic compounds using the Folin–Ciocalteu assay. The tannin concentration was calculated as the difference between total phenolic and non‐tannin phenolic contents and expressed as mg GAE/gram dry extract. All analyses were performed in triplicate in three independent experiments conducted on different days.

### 5.4 Mass Spectrometry (ESI‐FT‐ICR MS)

Fourier transform ion cyclotron resonance mass spectrometry (FT‐ICR MS) combined with a direct infusion electrospray ionization (ESI) provided a detailed molecular view of geopropolis extracts. The samples were dissolved in 1 mL of methanol and analyzed by high‐resolution mass spectrometry (HRMS). The mass spectrometer was the 9.4 T Solarix (Bruker Daltonics, Bremen, Germany). The analyzes were performed in the electrospray source and the negative ion mode, ESI(‐), over a mass range of *m/z* 150–1500. The ESI source conditions were as follows: a nebulizer gas pressure of 1.5 bar, a capillary voltage of 4.0–4.4 kV, and a capillary transfer temperature of 200° C. Ion time accumulation was 0.005–0.030 s. The time of flight was 0.950 sec. ESI(‐)FT‐ICR mass spectra were acquired by accumulating 16 scans of time‐domain transient signals in four mega‐point time‐domain data sets. All mass spectra were externally calibrated using Arginine (*m/z* from 150 to 1500). A mass accuracy of <10 ppm provided the unambiguous molecular formula assignments for singly charged molecular ions. Mass spectra were processed using data analysis software (Bruker Dantonics, Bremen, Germany). Elemental compositions of the compounds were determined by measuring the mass‐to‐charge ratio (*m/z*), considering the error and double bond equivalents (DBE) values [40]. The structural formulas of the compounds were obtained by ChemSpider software and compared with compounds frequently described in the scientific literature for bee products.

### 5.5 DPPH and ABTS•^+^ Radical Scavenging Assay

The antioxidant activity of the geopropolis extracts was evaluated using two complementary free radical scavenging assays based on the reduction of 2,2‐diphenyl‐1‐picrylhydrazyl (DPPH) and 2,2′‐azinobis‐(3‐ethylbenzothiazoline‐6‐sulfonic acid) radical cation (ABTS•^+^) [37]. In both assays, the antioxidant capacity of the extracts tested at concentrations ranging from to 1 to 800 µg/mL, was determined by measuring their ability to scavenge the stable free radicals in a concentration‐dependent manner. For the DPPH assay, absorbance was measured at 517 nm, whereas for the ABTS•^+^ assay, absorbance was measured at 734 nm using a SpectraMax 190 microplate spectrophotometer (Molecular Devices, Sunnyvale, CA, USA). The results were expressed as the half‐maximal inhibitory concentration (IC_50_, µg/mL), corresponding to the extract concentration required to reduce the initial radical concentration by 50%. Quercetin was used as the reference antioxidant for comparison. All experiments were performed in triplicate and independently repeated on three different days.

### 5.6 Cell Lines

Murine fibroblasts L929 (NCTC clone 929; RRID:CVCL‐0462) and RAW 264.7 macrophages (ATCC TIB‐71; RRID:CVCL‐0493) were obtained from the Rio de Janeiro Cell Bank (BCRJ, Rio de Janeiro, Brazil). Both cell lines were maintained in Dulbecco's Modified Eagle Medium (DMEM; Sigma‐Aldrich, St. Louis, MO, USA) supplemented with 10% fetal bovine serum (FBS; Sigma‐Aldrich, St. Louis, MO, USA), 100 IU/mL penicillin, and 100 µg/mL streptomycin. Cell cultures were incubated at 37 °C in a humidified atmosphere containing 5% CO_2_.

### 5.7 Cell Viability

Cell viability was evaluated using the colorimetric 3‐(4,5‐dimethylthiazol‐2‐yl)‐2,5‐diphenyltetrazolium bromide (MTT) assay [41]. Briefly, RAW 264.7 macrophages and L929 fibroblasts were seeded into 96‐well micro plates at a density of 1.2 × 10^4^ cells per well and allowed to adhere overnight under standard culture conditions. After the incubation period, the culture medium was replaced with fresh medium containing different concentrations of the geopropolis extracts (0.1‐150 µg/mL), and the cells were incubated for 24 h at 37 °C in a humidified atmosphere containing 5% CO_2_. Following treatment, MTT solution (1 mg/mL) was added to each well, and the plates were incubated for 2 h at 37 °C to allow the formation of intracellular formazan crystals by metabolically active cells. The resulting crystals were subsequently dissolved in dimethyl sulfoxide (DMSO), and the absorbance was measured at 595 nm (Molecular Devices, San Jose, CA, USA). Cell viability was expressed as the percentage of viable cells relative to the untreated control group. All experiments were performed in triplicate in three independent experiments.

### 5.8 Determination of Nitric Oxide Production

Nitric oxide (NO) production was determined indirectly by measuring nitrite accumulation in the culture supernatant using the Griess reaction [42]. Briefly, RAW 264.7 macrophages were seeded into 96‐well microplates at a density of 1.2 × 10^4^ cells per well and allowed to adhere overnight at 37 °C in a humidified atmosphere containing 5% CO_2_. The cells were then treated with geopropolis extracts at concentrations ranging from 0.1 to 20 µg/mL. After 1 h of treatment, lipopolysaccharide (LPS; 1 µg/mL) was added to all wells, except those of the negative control group, to induce the inflammatory response. L‐N^6^‐(1‐iminoethyl)lysine (L‐NIL; 30 µM), a selective inducible nitric oxide synthase (iNOS) inhibitor, was used as the positive control. Following 20 h of incubation, 50 µL of each culture supernatant was transferred to a new 96‐well plate and mixed with an equal volume (50 µL) of Griess reagent. Absorbance was measured using a SpectraMax 190 microplate spectrophotometer (Molecular Devices, Sunnyvale, CA, USA) at 540 nm. Nitrite concentration, used as an indicator of nitric oxide production, was determined from a sodium nitrite standard curve by linear regression analysis. The results were expressed as mean ± standard deviation (SD) of nitrite concentration (µM). All experiments were performed in triplicate in three independent experiments.

### 5.9 Superoxide Anion Production

The inhibitory effect of the geopropolis extracts on superoxide anion (O_2_•^−^) production was evaluated in lipopolysaccharide (LPS)‐stimulated RAW 264.7 macrophages, following the method described by Soares et al. (2021). Briefly, RAW 264.7 macrophages were seeded into 96‐well micro plates at a density of 1.2 × 10^4^ cells per well and allowed to adhere overnight under standard culture conditions (37 °C in a humidified atmosphere containing 5% CO_2_). The cells were then treated with geopropolis extracts at concentrations ranging from 0.1 to 20 µg/mL. After 1 h of treatment, LPS (1 µg/mL) was added to induce superoxide anion production. Tempol (4‐hydroxy‐2,2,6,6‐tetramethylpiperidine‐1‐oxyl; 10 µM) was used as the positive control. Superoxide anion production was determined using the nitroblue tetrazolium (NBT) reduction assay. Absorbance was measured at 630 nm using a SpectraMax 190 microplate spectrophotometer (Molecular Devices, Sunnyvale, CA, USA). The results were expressed as the percentage of superoxide anion production relative to the LPS‐stimulated control group (mean ± standard deviation, SD). All experiments were performed in triplicate in at least three independent experiments.

### 5.10 Measurement of Cytokines

Cell treatment was performed as described for the nitric oxide production assay. The concentration of tumor necrosis factor‐alpha (TNF‐α) in the culture supernatants was quantified using a commercial enzyme‐linked immunosorbent assay (ELISA) kit (eBioscience, San Diego, CA, USA), according to the manufacturer's instructions. Absorbance was measured at 450 nm using a SpectraMax 190 microplate spectrophotometer (Molecular Devices, Sunnyvale, CA, USA), and cytokine concentrations were calculated from a recombinant murine TNF‐α standard curve. Results were expressed as mean ± standard deviation (SD) from three independent experiments performed in triplicate.

### 5.11 Antifungal Activity

Antifungal activity was evaluated against the reference strains A*. fumigatus* ATCC 16913, T*. rubrum* CBS 392.58, C. *albicans* ATCC 10231, C*. albicans* ATCC 90029, and C. *parapsilosis* ATCC 22019 using the broth microdilution method in 96‐well microplates. MICs were determined according to the Clinical and Laboratory Standards Institute (CLSI) guidelines M27‐A3 and M38‐A. Geopropolis extracts were dissolved in dimethyl sulfoxide (DMSO) and diluted in RPMI‐1640 medium buffered with MOPS to obtain final concentrations ranging from 8 to 1024 µg/mL. Fluconazole and Amphotericin B (0.03–16 µg/mL) were used as reference antifungal drugs. MIC was defined as the lowest concentration showing no visible fungal growth after incubation for 24 h (*Candida* spp.), 48 h (A*. fumigatus*), or 96 h (T*. rubrum*). *Aspergillus flavus* ATCC 204304 was included as the quality control strain. MFCs were determined according to the method of Espinel‐Ingroff et al. (2002). Aliquots (20 µL) from wells corresponding to the MIC, 2 × MIC, and 4 × MIC were subculture onto Sabouraud dextrose agar and incubated under the same conditions described above. The MFC was defined as the lowest concentration that prevented visible fungal growth, corresponding to approximately 99%–99.5% fungal killing [43].

### 5.12 Statistical Analysis

Data are presented as mean ± standard deviation (SD). Statistical analyses were performed using R software (R Foundation for Statistical Computing, Vienna, Austria; version 3.1.1). Differences among groups were evaluated by two‐way analysis of variance (ANOVA), followed by Tukey's multiple comparison test. Differences were considered statistically significant at *p* < 0.05.

## Author Contributions

**Ariane Pinheiro Cruz Bergamini**: Conceptualization, formal analysis, investigation, data curation, and Writing – original draft. **Antônio Domingos de Sousa Júnior**, **Flavia Vitorino de Araújo Porto**, **Iana Soares Pessoa**, **Thiago Antonio de Sousa Cutrim**, **Victor da Rocha Fonseca**: Investigation and validation. **Victor Paulo Mesquita Aragão**: Formal analysis and validation. **Wanderson Romão**, **Denise Coutinho Endringer**, **Rodrigo Scherer**: Methodology and validation. **Marcio Fronza**: Conceptualization, methodology, validation, resources, writing – review & editing, supervision, project administration, and funding acquisition. All authors have read and agreed to the published version of the manuscript.

## Declaration of Generative AI and AI‐Assisted Technologies in the Writing Process

During the preparation of this manuscript, the authors used ChatGPT‐4 and Microsoft 365 Copilot, for English language editing and grammar correction to improve the clarity and readability of the text. After using this tool/service, the author(s) reviewed and edited the content as needed and takes full responsibility for the content of the publication.

## Conflicts of Interest

The authors declare no conflicts of interest.

## Supporting information




**Supporting File 1**: cbdv71615‐sup‐0001‐SuppMat.docx

## Data Availability

The data that support the findings of this study are available on request from the corresponding author. The data are not publicly available due to privacy or ethical restrictions.
