# Supplementary material for: Chemical Composition and Biological Activities of Geopropolis From Four Melipona Species From the Brazilian Atlantic Forest
Source: Chem Biodivers. 2026 Aug 25;23(8):e71615. doi: 10.1002/cbdv.71615 (PMC13506092; doi:10.1002/cbdv.71615)
Supplement: Supplementary file 1 [file CBDV-23-e71615-s001.docx]

**Chemical composition and biological activities of geopropolis from four *Melipona* species from the Brazilian Atlantic Forest**

Ariane Pinheiro Cruz Bergamini^a^, Antônio Domingos de Sousa Júnior ^a^, Flavia Vitorino de Araújo Porto^a^, Iana Soares Pessoa^a^, Thiago Antonio de Sousa Cutrim^a^, Victor Paulo Mesquita Aragão^a^, Victor da Rocha Fonseca^b^, Wanderson Romão^b^, Denise Coutinho Endringer^a^, Rodrigo Scherer ^a^, Marcio Fronza^a^*

**SUPPLEMENTAL ONLINE MATERIAL**

**Figure 1S.** Representative ESI(-)-FT-ICR MS spectra of hydroalcoholic geopropolis extracts obtained from *Melipona quadrifasciata* (MQ), *Melipona mondury* (MM), *Melipona capixaba* (MC), and *Melipona bicolor* (MB) collected during the rainy and dry seasons. The spectra illustrate the overall metabolite profiles and the relative abundance of the major ions detected in each sample.

**Table 1S.** Tentatively annotated chemical compounds detected in hydroalcoholic geopropolis extracts from four stingless bee species (*Melipona quadrifasciata* (MQ), *Melipona mondury* (MM), *Melipona capixaba* (MC), and *Melipona bicolor* (MB)) collected during the rainy and dry seasons and analyzed by ESI(−)-FT-ICR MS. Compound annotation was based on accurate mass measurements, molecular formula prediction, double bond equivalents (DBE), mass error (≤10 ppm), and comparison with previously reported compounds in the literature. D = detected; ND = not detected.

| N° | **m/z (exper)** | **Molecular Formulas [M-H]-** | **DBE** | **error (ppm)** | **Structural Formulas** | **MQ**  **Rainy** | **MQ**  **Dry** | **MM**  **Rainy** | **MM**  **Dry** | **MC**  **Rainy** | **MC**  **Dry** | **MB**  **Rainy** | **MB**  **Dry** | **Reference** |
| --- | --- | --- | --- | --- | --- | --- | --- | --- | --- | --- | --- | --- | --- | --- |
| 1 | 179.05636 | C_6_ H_11_O_6_ | 1 | -0.21 | Glicose | ND | ND | ND | ND | D | ND | ND | ND | [1,2] |
| 2 | 181.05066 | C_9_H_9_O_4_ | 5 | 1.24 | Syringaldehyde | ND | ND | ND | ND | D | ND | ND | ND | [3] |
| 3 | 191.05618 | C_7_H_11_O_6_ | 2 | 7.76 | Quinic acid | ND | ND | ND | ND | D | ND | ND | ND | [4] |
| 4 | 193.04973 | C_10_H_9_O_4_ | 6 | 4.83 | Ferulic acid | ND | ND | ND | ND | D | D | ND | ND | [5,6] |
| 5 | 195.05105 | C_6_H_11_O_7_ | 1 | 2.43 | Gluconic acid | ND | ND | ND | ND | D | D | D | D | [3,7,8] |
| 6 | 197.04563 | C_9_H_9_O_5_ | 5 | 8.21 | Syringic acid | D | D | ND | ND | D | ND | D | ND | [3,8] |
| 7 | 203.17996 | C_15_H_23_ | 4 | -5.10 | Copaene / Bergamotene / Guaiene / Aromadendrene / Himachalene / Humulene / Farnesene / Caryophyllene / muurolene / Germacrene / Chamigrene / Selinene / Valencene / Bisabolene / Cadinene | D | D | D | D | D | D | D | D | [9] |
| 8 | 207.06619 | C_11_H_11_O_4_ | 6 | 6.60 | Sinapaldehyde | D | D | ND | ND | D | D | ND | D | [3] |
| 9 | 223.05994 | C_11_H_11_O_5_ | 6 | 5.64 | sinapic ac | ND | ND | D | D | ND | D | ND | D | [3] |
| 10 | 231.10276 | C_14_H_15_O_3_ | 7 | 7.95 | Drupanin / [hydroxycinnamic acid](http://www.molbase.com/cas/78834-99-8.html) | ND | ND | D | ND | ND | ND | ND | D | [10] |
| 11 | 247.09771 | C_14_H_15_O_4_ | 7 | 8.27 | Prenyl caffeate / Prenylated caffeic acid | D | ND | D | ND | ND | ND | D | D | [10] |
| 12 | 253.04847 | C_15_H_9_O_4_ | 11 | -9.99 | Chrysin | D | ND | D | D | ND | D | D | D | [3,4,11] |
| 13 | 255.23296 | C_16_H_31_O_2_ | 1 | -8.31 | Acide palmitique | D | D | D | D | D | D | D | D | [3,4] |
| 14 | 263.1280 | C_15_H_19_O_4_ | 4 | 9.82 | Abscisic acid | D | D | D | D | D | D | D | D | [6] |
| 15 | 269.0455 | C_15_H_9_O_5_ | 11 | -9.08 | Apigenin / Galangin | D | D | ND | ND | ND | D | D | D | [3,5,6,12] |
| 16 | 269.080 | C_16_H_13_O_4_ | 8 | 8.26 | Medicarpin | ND | ND | D | D | D | D | D | D | [11] |
| 17 | 271.0612 | C_15_H_11_O_5_ | 11 | -10.01 | naringenin | D | D | D | D | D | D | D | D | [3,8,13] |
| 18 | 277.21742 | C_18_H_29_O_2_ | 4 | 7.61 | Linolenic acid | D | D | D | D | D | ND | ND | ND | [7] |
| 19 | 279.23306 | C_18_H_31_O_2_ | 3 | -9.27 | Linoleic acid | D | D | D | D | ND | D | D | D | [7] |
| 20 | 281.24878 | C_18_H_33_O_2_ | 2 | -0.62 | Oleic acid | ND | ND | D  0,07 | D | D  -0,62 | D  -0,42 | D  -0,12 | D  0,07 | NR |
| 21 | 283.26442 | C_18_H_35_O_2_ | 1 | -7.79 | Stearic acid | D | D | D | D | ND | D | D | D | [4] |
| 22 | 285.2316 | C_20_H_29_O | 6 | -9.92 | Vitamin A / Retinol | D | D | ND | ND | ND | ND | D | ND | [1] |
| 23 | 285.04051 | C_15_H_9_O_6_ | 11 | -2.87 | Kaempferol / Luteolin | ND | ND | D | D | D | D | D | ND | [1,3,7,12,14–16] |
| 24 | 287.05611 | C_15_H_11_O_6_ | 10 | 9.72 | Plathymenin / Eriodictyol / Aromadendrin | D | D | D | D | D | D | D | D | [11] |
| 25 | 289.06978 | C_15_H_13_O_6_ | 9 | 9.35 | Catechin / Epicatechin | D | D | D | D | D | D | D | D | [3,8,15] |
| 26 | 299.05618 | C_16_H_11_O_6_ | 11 | -5.71 | Hispidulin | D | D | ND | ND | D | ND | ND | D | [3] |
| 27 | 300.99926 | C_14_H_5_O_8_ | 12 | -4.15 | Ellagic acid | D | D | D | D | ND | D | D | ND | [3,7,8,14,15,17,18] |
| 28 | 301.03569 | C_15_H_9_O_7_ | 11 | 9.94 | Quercetin / Tricetin | ND | ND | ND | ND | D | D | D | D | [3,5,17,18] |
| 29 | 301.07194 | C_16_H_13_O_6_ | 10 | 5.95 | Isoferreirina / Alnustinol / Ferreirina / Hesperetin | D | ND | D | ND | D | ND | D | D | [15] |
| 30 | 303.05118 | C_15_H_11_O_7_ | 10 | 9.92 | Taxifolin / Dihydroquercetin | D | D | ND | ND | D | ND | ND | ND | [3] |
| 31 | 315.05126 | C_16_H_11_O_7_ | 11 | 7.73 | Isorhamnetin / Selagin / Methoxyherbacetin | D | D | D | D | D | D | D | D | [12,19] |
| 32 | 317.03032 | C_15_H_9_O_8_ | 11 | -4.02 | Myricetin | ND | ND | D | D | ND | ND | D | ND | [3] |
| 33 | 329.17604 | C_20_H_25_O_4_ | 8 | 9.94 | carnosol | D | ND | D | ND | ND | D | D | ND | [10]  [3] |
| 34 | 331.06632 | C_13_H_15_O_10_ | 6 | -7.49 | monogalloylglucose | D | D | D | D | D | D | D | D | [14] |
| 35 | 353.08788 | C_16_H_17_O_9_ | 8 | -8.00 | Chlorogenic acid / caffeoylquinic acid | D | D | D | D | D | D | D | D | [3,6,10] |
| 36 | 359.07581 | C_18_H_15_O_8_ | 11 | 9.47 | Rosmarinic acid | D | D | ND | ND | D | D | ND | ND | [3] |
| 37 | 405.17089 | C_25_H_25_O_5_ | 13 | -0.35 | diprenilgenisteina | D | D | D | D | D | D | D | D | [9] |
| 38 | 407.20102 | C_22_H_31_O_21_ | 15 | 9.40 | Galloyl pinocembrin | D | D | D | D | ND | ND | D | D | [13] |
| 39 | 409.172943 | C_24_H_25_O_6_ | 11 | -5.35 | Mangostin | D | D | ND | ND | ND | D | ND | D | [13,20] |
| 40 | 413.37882 | C_29_H_49_O | 5 | -9.66 | Sitosterol | D | D | D | D | D | D | D | ND | [5,9,19] |
| 41 | 421.16576 | C_25_H_25_O_6_ | 13 | -0.75 | Diprenilkaempferol / Cajanona | D | D | D | D | D | D | D | D | [9,21] |
| 42 | 421.084900 | C_19_H_17_O_11_ | 11 | 6.35 | Mangiferin | D | D | D | D | D | D | D | D | [13] |
| 43 | 423.07106 | C_22_H_15_O_9_ | 15 | 9.80 | Galloyl naringenin | D | D | D | D | ND | D | D | ND | [13] |
| 44 | 431.09859 | C_21_H_19_O_10_ | 12 | 6.91 | apigenin-glucoside | D | D | D | D | D | D | D | ND | [14] |
| 45 | 439.0639 | C_22_H_15_O_10_ | 15 | 9.84 | Galloyl eriodictyol | D | D | D | D | D | D | D | D | [13] |
| 46 | 447.09331 | C_21_H_19_O_11_ | 12 | -10.03 | quercitrin | D | D | ND | ND | D | D | D | D | NR |
| 47 | 463.08836 | C_21_H_19_O_12_ | 12 | 8.94 | Isoquercetin | D | D | D | D | D | D | D | D | [3] |
| 48 | 477.07226 | C_21_H_17_O_13_ | 13 | 0.66 | digalloylshikimic acid | D | D | D | D | D | D | D | D | [22] |
| 49 | 501.30110 | C_33_H_41_O_4_ | 13 | -0.14 | Nemorosone / Propolone A | D | D | D | D | D | D | D | D | [9] |
| 50 | 515.11991 | C_25_H_23_O_12_ | 14 | 3.10 | caffeoylquinic acid | ND | ND | ND | ND | D | ND | ND | ND | [10] |
| 51 | 579.13571 | C_26_H_27_O_15_ | 13 | -5.74 | Carlinoside | D | D | D | D | D | D | D | D | NR |
| 52 | 579.16647 | C_27_H_31_O_14_ | 12 | -4.33 | Naringin | D | D | D | D | D | D | D | D | [3,6] |
| 53 | 603.00535 | C_28_H_11_O_16_ | 23 | 0.78 | ellagic acid dimer | D | D | D | D | ND | D | D | ND | [7] |
| 54 | 609.12554 | C_30_H_25_O_14_ | 18 | 8.75 | prodelphinidin B4 | ND | ND | ND | ND | D | D | ND | ND | NR |
| 55 | 609.1478 | C_27_H_29_O_16_ | 13 | -2.79 | Rutin | D | D | D | D | D | D | D | D | [3,7,8,15,18] |
| 56 | 625.1412 | C_27_H_29_O_17_ | 13 | -7.95 | quercetin-diglucoside | D | D | D | D | D | D | D | D | [7,14,18] |

References

[1]	J.F. Campos, U.P. dos Santos, P. dos S. da Rocha, M.J. Damião, J.B.P. Balestieri, C.A.L. Cardoso, E.J. Paredes-Gamero, L.M. Estevinho, K. de Picoli Souza, E.L. dos Santos, Antimicrobial, Antioxidant, Anti-Inflammatory, and Cytotoxic Activities of Propolis from the Stingless Bee Tetragonisca fiebrigi (Jataí), Evidence-Based Complement. Altern. Med. 2015 (2015) 1–11. https://doi.org/10.1155/2015/296186.

[2]	R.P. Dutra, B.V. De Barros Abreu, M.S. Cunha, M.C.A. Batista, L.M.B. Torres, F.R.F. Nascimento, M.N.S. Ribeiro, R.N.M. Guerra, Phenolic acids, hydrolyzable tannins, and antioxidant activity of geopropolis from the stingless bee Melipona fasciculata smith, J. Agric. Food Chem. 62 (2014) 2549–2557. https://doi.org/10.1021/jf404875v.

[3]	B.L. Ferreira, L.V. Gonzaga, L. Vitali, G.A. Micke, D. Baggio, A.C. de Oliveira Costa, R. Fett, Dataset about Southern-Brazilian geopropolis: Physical and chemical perspectives, Data Br. 29 (2020) 105109. https://doi.org/10.1016/j.dib.2020.105109.

[4]	R.P. Dutra, J.L. Bezerra, M.C.P. da Silva, M.C.A. Batista, F.J.B. Patrício, F.R.F. Nascimento, M.N.S. Ribeiro, R.N.M. Guerra, Antileishmanial activity and chemical composition from Brazilian geopropolis produced by stingless bee Melipona fasciculata, Rev. Bras. Farmacogn. 29 (2019) 287–293. https://doi.org/10.1016/j.bjp.2019.02.009.

[5]	T. Bonamigo, J.F. Campos, T.M. Alfredo, J.B.P. Balestieri, C.A.L. Cardoso, E.J. Paredes-Gamero, K. de Picoli Souza, E.L. Dos Santos, Antioxidant, Cytotoxic, and Toxic Activities of Propolis from Two Native Bees in Brazil: Scaptotrigona depilis and Melipona quadrifasciata anthidioides., Oxid. Med. Cell. Longev. 2017 (2017) 1038153. https://doi.org/10.1155/2017/1038153.

[6]	R.G. de Oliveira, S. Jain, L. dos S. Freitas, E.D. de Araújo, Phenolic compound, nutritional and antioxidant profile of pollen collected by the genus melipona in North Eastern Brazil, Brazilian J. Food Technol. 22 (2019). https://doi.org/10.1590/1981-6723.07918.

[7]	A.J.O. Lopes, C.C. Vasconcelos, F.A.N. Pereira, R.H.M. Silva, P.F.D.S.Q. Queiroz, C.V. Fernandes, J.B.S. Garcia, R.M. Ramos, C.Q. Da Rocha, S.T.D.J.R.M. Lima, M.D.S.D.S. Cartágenes, M.N. De Sousa Ribeiro, Anti-Inflammatory and antinociceptive activity of pollen extract collected by stingless bee Melipona fasciculata, Int. J. Mol. Sci. 20 (2019) 4512. https://doi.org/10.3390/ijms20184512.

[8]	T.L.A. DOS Santos, R.F. Queiroz, A.C.H.F. Sawaya, B.G.-C. Lopez, M.B.P. Soares, D.P. Bezerra, A.C.B.C. Rodrigues, V.F.D. Paula, A.M. Waldshmidt, Melipona mondury produces a geopropolis with antioxidant, antibacterial and antiproliferative activities, An. Acad. Bras. Cienc. 89 (2017) 2247–2259. https://doi.org/10.1590/0001-3765201720160725.

[9]	E. Souza, E. Silva, H. Cordeiro, N. Lage Filho, F. Silva, D. Reis, C. Porto, E. Pilau, L.A. Costa, A. Souza, C. Menezes, A. Flach, Chemical compositions and antioxidant and antimicrobial activities of propolis produced by Frieseomelitta longipes and Apis mellifera bees, Quim. Nova 41 (2018) 485–491. https://doi.org/10.21577/0100-4042.20170208.

[10]	D.S. de Oliveira Dembogurski, D. Silva Trentin, A.G. Boaretto, G.V. Rigo, R.C. da Silva, T. Tasca, A.J. Macedo, C.A. Carollo, D.B. Silva, Brown propolis-metabolomic innovative approach to determine compounds capable of killing Staphylococcus aureus biofilm and Trichomonas vaginalis, Food Res. Int. 111 (2018) 661–673. https://doi.org/10.1016/j.foodres.2018.05.033.

[11]	M. Funakoshi-Tago, K. Ohsawa, T. Ishikawa, F. Nakamura, F. Ueda, Y. Narukawa, F. Kiuchi, H. Tamura, K. Tago, T. Kasahara, Inhibitory effects of flavonoids extracted from Nepalese propolis on the LPS signaling pathway, Int. Immunopharmacol. 40 (2016) 550–560. https://doi.org/10.1016/j.intimp.2016.10.008.

[12]	Z.A. Othman, W.S. Wan Ghazali, L. Noordin, N.A. Mohd. Yusof, M. Mohamed, Phenolic Compounds and the Anti-Atherogenic Effect of Bee Bread in High-Fat Diet-Induced Obese Rats, Antioxidants 9 (2019) 33. https://doi.org/10.3390/antiox9010033.

[13]	F.C. Chong, L.S. Chua, Effects of Solvent and pH on Stingless Bee Propolis in Ultrasound-Assisted Extraction, AgriEngineering 2 (2020) 308–316. https://doi.org/10.3390/agriengineering2020020.

[14]	A.J.O. Lopes, C.C. Vasconcelos, J.B.S. Garcia, M.S.D. Pinheiro, F.A.N. Pereira, D. de S. Camelo, S.V. de Morais, J.R.B. Freitas, C.Q. da Rocha, M.N. de S. Ribeiro, M. do S. de S. Cartágenes, Anti-Inflammatory and Antioxidant Activity of Pollen Extract Collected by Scaptotrigona affinis postica: in silico, in vitro, and in vivo Studies, Antioxidants 9 (2020) 103. https://doi.org/10.3390/antiox9020103.

[15]	M.J.A.M. Araújo, S. de M.G. Bosco, J.M. Sforcin, Pythium insidiosum: inhibitory effects of propolis and geopropolis on hyphal growth, Brazilian J. Microbiol. 47 (2016) 863–869. https://doi.org/10.1016/j.bjm.2016.06.008.

[16]	J.H. de O. Reis, G. de A. Barreto, J.C. Cerqueira, J.P. dos Anjos, L.N. Andrade, F.F. Padilha, J.I. Druzian, B.A.S. Machado, Evaluation of the antioxidant profile and cytotoxic activity of red propolis extracts from different regions of northeastern Brazil obtained by conventional and ultrasound-assisted extraction., PLoS One 14 (2019) e0219063. https://doi.org/10.1371/journal.pone.0219063.

[17]	S. Alves De Souza, C.A. Camara, E. Monica Sarmento Da Silva, T.M.S. Silva, Composition and antioxidant activity of geopropolis collected by Melipona subnitida (jandaíra) bees, Evidence-Based Complement. Altern. Med. 2013 (2013) 1–5. https://doi.org/10.1155/2013/801383.

[18]	M.D. Belina-Aldemita, M. Schreiner, S. D’Amico, Characterization of phenolic compounds and antioxidative potential of pot-pollen produced by stingless bees (Tetragonula biroi Friese) from the Philippines., J. Food Biochem. 44 (2020) e13102. https://doi.org/10.1111/jfbc.13102.

[19]	T.M.S. Silva, C.A. Camara, A.C. da Silva Lins, J. Maria Barbosa-Filho, E.M.S. da Silva, B.M. Freitas, F. de Assis Ribeiro dos Santos, Chemical composition and free radical scavenging activity of pollen loads from stingless bee Melipona subnitida Ducke, J. Food Compos. Anal. 19 (2006) 507–511. https://doi.org/10.1016/j.jfca.2005.12.011.

[20]	B. Vongsak, S. Kongkiatpaiboon, S. Jaisamut, S. Machana, C. Pattarapanich, In vitro alpha glucosidase inhibition and free-radical scavenging activity of propolis from Thai stingless bees in mangosteen orchard, Rev. Bras. Farmacogn. 25 (2015) 445–450. https://doi.org/10.1016/J.BJP.2015.07.004.

[21]	A.W.F. DUARTE, M.R. dos S. VASCONCELOS, M. ODA-SOUZA, F.F. de OLIVEIRA, A.M.Q. LÓPEZ, Honey and bee pollen produced by Meliponini (Apidae) in Alagoas, Brazil: multivariate analysis of physicochemical and antioxidant profiles, Food Sci. Technol. 38 (2018) 493–503. https://doi.org/10.1590/fst.09317.

[22]	M. Sousa Gomes, R. Diógenes Alves Uchoa Lins, S.M. Zucolotto Langassner, É.J. Dantas da Silveira, T. Gomes de Carvalho, M.L. Diniz de Sousa Lopes, L. de Souza Araújo, C. Addison Carvalho Xavier de Medeiros, R. Ferreira de Carvalho Leitão, G. Coelho Bernardo Guerra, R. Fernandes de Araújo Junior, A. Antunes de Araújo, Anti-inflammatory and antioxidant activity of hydroethanolic extract of Spondias mombin leaf in an oral mucositis experimental model, Arch. Oral Biol. 111 (2020) 104664. https://doi.org/10.1016/j.archoralbio.2020.104664.
